# Small-Molecule Modulators of Sigma1 and Sigma2/TMEM97 in the Context of Cancer: Foundational Concepts and Emerging Themes

**DOI:** 10.3389/fphar.2019.01141

**Published:** 2019-10-21

**Authors:** Halley M. Oyer, Christina M. Sanders, Felix J. Kim

**Affiliations:** Department of Cancer Biology, Sidney Kimmel Cancer Center at Thomas Jefferson University, Philadelphia, PA, United States

**Keywords:** Sigma1, Sigma2/TMEM97, cancer, pharmacology, autophagy, proteostasis, metabolism

## Abstract

There are two known subtypes of the so-called sigma receptors, Sigma1 and Sigma2. Sigma1 (encoded by the *SIGMAR1* gene and also known as Sigma-1 receptor, S1R) is a unique pharmacologically regulated integral membrane chaperone or scaffolding protein that allosterically modulates the activity of its associated proteins. Sigma2, recently identified as transmembrane protein 97 (TMEM97), is an integral membrane protein implicated in cellular cholesterol homeostasis. A number of publications over the past two decades have suggested a role for both sigma proteins in tumor biology. Although there is currently no clinically used anti-cancer drug that targets Sigma1 or Sigma2/TMEM97, a growing body of evidence supports the potential of small-molecule compounds with affinity for these proteins, putative sigma ligands, as therapeutic agents to treat cancer. In preclinical models, these compounds have been reported to inhibit cancer cell proliferation, survival, adhesion, and migration; furthermore, they have been demonstrated to suppress tumor growth, to alleviate cancer-associated pain, and to exert immunomodulatory properties. Here, we will address the known knowns and the known unknowns of Sigma1 and Sigma2/TMEM97 ligand actions in the context of cancer. This review will highlight key discoveries and published evidence in support of a role for sigma proteins in cancer and will discuss several fundamental questions regarding the physiological roles of sigma proteins in cancer and sigma ligand mechanism of action.

## Discovery, Rediscovery, and Identification of Sigma1 and Sigma2/TMEM97 Receptors

The notion of sigma receptors began with the discovery of the Sigma1-binding site in 1976 (Martin et al., 1976). In this study, three distinct classes of opioid receptors, mu, kappa, and sigma were proposed based upon behavioral studies with morphine, ketocyclazocine, and SKF10047. The opioid receptor antagonist naltrexone antagonized all of these compounds, which led to the identification of sigma as an opioid receptor (Martin et al., 1976). However, in the original study, the stereoisomer of SKF10047 used was not described. Subsequent studies used (+)-SKF10047 to define the putative sigma receptor as clearly not opioid (Su, 1982). Since then, a large number of chemically diverse compounds that have affinity for sigma receptors have been reported (reviewed in Cobos EJ et al., 2008; Maurice and Su, 2009; Narayanan et al., 2011; Weber and Wunsch, 2017). Based primarily on ligand-binding studies with this growing number of compounds, the putative sigma receptors were subdivided into two subtypes, Sigma1 and Sigma2 (Hellewell and Bowen, 1990).

Sigma1 (*SIGMAR1*; also known as Sigma1-receptor and several other names (Kim, 2017) has been more extensively characterized than Sigma2. The cloning of Sigma1 revealed that it was unlike any traditional receptor (Hanner et al., 1996). Indeed, Sigma1 shares no significant homology with any other protein encoded in the human genome (Hanner et al., 1996; Schmidt et al., 2016). Full-length human Sigma1 is an approximately 26 kilodalton (kDa) protein that comprises 223 amino acids. According to the recently published crystal structure, Sigma1 has a single integral membrane domain with a short ER luminal amino-terminal peptide and most of the carboxy-terminal region of the protein extending into the cytoplasm (Hanner et al., 1996; Schmidt et al., 2016). Emerging evidence suggests that Sigma1 is a novel, pharmacologically responsive, oligomeric, and integral membrane chaperone or scaffolding protein (Hayashi and Su, 2007; Crottes et al., 2011; Crottes et al., 2016; Thomas et al., 2017) that is enriched in the secretory pathway, particularly the endoplasmic reticulum (ER) of most cancer cells (reviewed in Kim and Maher, 2017). In the context of tumor biology, Sigma1 appears to be a component of the cancer cell support machinery (Kim and Maher, 2017). Sigma1 has been proposed to function as oligomeric structures including dimers, trimers, tetramers, and higher order oligomers (Gromek et al., 2014; Schmidt et al., 2016). Changes in oligomeric structures may correspond with differential response to Sigma1 ligands (Gromek et al., 2014; Mishra et al., 2015; Schmidt et al., 2018; Yano et al., 2018). Although the label “receptor” persists, it is now clear that Sigma1 does not fit the traditional definition of receptor. Sigma1 itself has no known intrinsic signaling or enzymatic activity, rather it allosterically modulates the intracellular signaling and activities of its associated proteins (reviewed in Maurice and Su, 2009; Kim and Maher, 2017; Pasternak, 2017).

Sigma2 had long remained a pharmacologically defined entity (Bowen, 2000; Zeng et al., 2017; Abate et al., 2018). Recently, the Sigma2 receptor was identified as an integral membrane protein called transmembrane protein 97 (TMEM97, also known as MAC30) (Alon et al., 2017; Kim and Pasternak, 2017), a member of the insulin-like growth factor-binding protein family (Murphy et al., 1993; Schmit and Michiels, 2018). TMEM97 has been implicated in cholesterol metabolism (Wilcox et al., 2007; Sanchez-Pulido and Ponting, 2014; Ebrahimi-Fakhari et al., 2016; Riad et al., 2018) and has been shown specifically to influence cellular cholesterol trafficking by binding to Niemann–Pick disease, type C1 (NPC1) protein (Bartz et al., 2009; Ebrahimi-Fakhari et al., 2016). TMEM97 also has been implicated in several types of cancer (Schmit and Michiels, 2018). The pharmacologically defined Sigma2-binding site has been implicated in myriad diseases and disorders, including cancer and neurodegenerative diseases (Wheeler et al., 2000; Crawford and Bowen, 2002; Crawford et al., 2002; Narayanan et al., 2011; Guo and Zhen, 2015; Abate et al., 2018). However, the molecular basis of these associations remains unclear. Validation of Sigma2/TMEM97 as the pharmacological target of Sigma2 ligands should enable molecular characterization of this sigma-binding site and open the door to more studies exploring the mechanism of action of putative sigma receptor ligands.

## Sigma1 and Sigma2/TMEM97 Expression in Cancer

Over the past two decades, a number of publications have suggested a potential role for Sigma1 (Kim and Maher, 2017) and Sigma2/TMEM97 (Abate et al., 2018; Schmit and Michiels, 2018) in tumor biology. Until recently, for Sigma1, this association was largely based on two lines of evidence: (Martin et al., 1976) elevated expression of *SIGMAR1* transcripts and Sigma1 protein, primarily in cancer cell lines and some tumors (Kim and Maher, 2017) and (Su, 1982) antiproliferative and apoptosis inducing effects of some small-molecule inhibitors (putative antagonists) of Sigma1 on cancer cell lines (reviewed extensively in (Kim and Maher, 2017) and briefly outlined in **Table 1**). The physiological significance of elevated Sigma1 in tumors remains poorly understood, and how *SIGMAR1* gene expression is regulated in cancer remains unclear. However, Sigma1 RNAi knockdown and some small-molecule inhibitors of Sigma1 inhibit cancer cell growth, proliferation, mobility, and survival and suppress xenografted tumor growth, suggesting that functional Sigma1 is required for tumorigenesis and tumor progression (Spruce et al., 2004; Sun et al., 2014; Kim and Maher, 2017; Thomas et al., 2017). Conversely, in some studies, increased Sigma1 protein levels through overexpression of recombinant Sigma1 and enhancing Sigma1 with small-molecule activators (putative agonists) have been reported to promote cell growth, proliferation, mobility, and cell survival (Zhu et al., 2003; Spruce et al., 2004; Maurice and Su, 2009; Sun et al., 2014; Thomas et al., 2017; Maher et al., 2018).

**Table 1:** Prototypical small-molecule Sigma1 and Sigma2/TMEM97 modulators/ligands.

Compound	Binding affinity (Sigma1 and 2) and references	Putative action	Assays used	Summary of results	References
(+)-Pentazocine 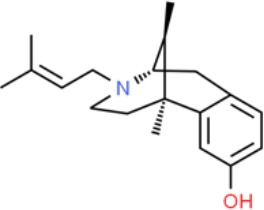	• Sigma1 (K_d_): 3.9–23.3 nM (Hellewell et al., 1994; John et al., 1999; Colabufo et al., 2004; Azzariti et al., 2006) • Sigma2 (K_i_): 1,542–6,611 nM (Hellewell et al., 1994; Vilner and Bowen, 2000; Choi et al., 2001; Ishiwata et al., 2006)	Agonist (Sigma1)	MTT, MTS, apoptosis assays, light microscopy of cell morphology changes	In most functional studies, it did not impact cell viability or proliferation, and it has been used to block the anticancer actions (cytotoxicity and/or proliferation arrest) of Sigma1 inhibitors/antagonists such as IPAG and rimcazole. In some cases, (+)-pentazocine reported to result in cell detachment and rounding of cells and inhibition of cell proliferation. (^3^H)(+)-pentazocine is a commonly used radioligand used to quantify and define Sigma1-binding sites.	(Brent and Pang, 1995; Colabufo et al., 2004; Spruce et al., 2004; Rybczynska et al., 2008; Korpis et al., 2014)
(+)-SKF10047 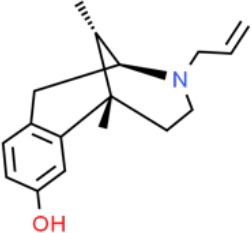	• Sigma1 (K_i_): 54–597 nM (Hellewell et al., 1994; Vilner et al., 1995a; Ryan-Moro et al., 1996; Vilner and Bowen, 2000) • Sigma2 K_i_: 11,170–39,740 nM (Hellewell et al., 1994; Vilner and Bowen, 2000)	Agonist (Sigma1)	MTT, MTS, or apoptosis assays, light microscopy of cell morphology changes	(+)-SKF10047 has been used to block the anticancer actions (cytotoxicity and/or proliferation arrest) of Sigma1 inhibitors/antagonists such as IPAG and rimcazole. Demonstrated immune modulatory effects by altering cytokine production as well as cytokine-induced signaling in tumor cells. In some cases, (+)-SKF10047 has been reported to result in cell detachment, rounding of cells, and inhibition of proliferation.	(Brent and Pang, 1995; Zhu et al., 2003; Spruce et al., 2004; Do et al., 2013)
BD1047 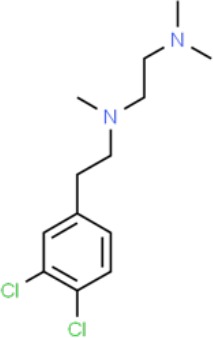	• Sigma1 K_i_: 0.6–5.3 nM (Matsumoto et al., 1995; Vilner et al., 1995a; Vilner et al., 1995b; Cobos et al., 2005; Entrena et al., 2009) • Sigma2 K_i_: 47 nM (Matsumoto et al., 1995)	Antagonist (Sigma1)	MTS, apoptosis assays, light microscopy of cell morphology changes, *in vivo* tumor model	Minimal anticancer activity, despite putative antagonist status (defined in behavioral assays). Induced altered cell morphology, but did not cause cancer death. Blocked antiproliferative and cytotoxic actions of Sigma2/TMEM97 ligands. Blocked PRE-084-induced tumor growth in immune competent mouse tumor implantation model.	(Vilner et al., 1995a; Moody et al., 2000; Zhu et al., 2003; Spruce et al., 2004; Kim and Maher, 2017)
CB-184 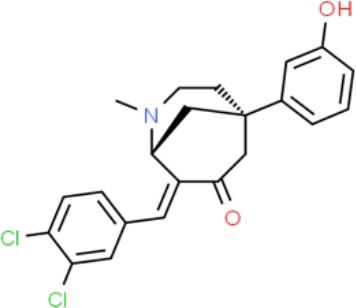	• Sigma1 K_i_: 7,436 nM (Bowen et al., 1995) • Sigma2 K_i_: 13 nM (Bowen et al., 1995)	Agonist (Sigma2/TMEM97)	MTT, LDH release, apoptosis assays	Cytotoxic effect in cancer cell line cultures as single agent. Potentiated cytotoxic chemotherapeutic agents actinomycin D and doxorubicin. Reported to trigger p53- and caspase- independent apoptosis.	(Bowen et al., 1995; Crawford and Bowen, 2002; Crawford et al., 2002)
DTG 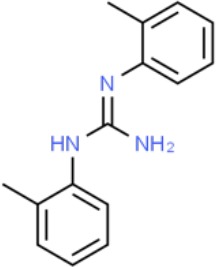	• Sigma1 K_i_: 45–203 nM (Hellewell et al., 1994; Vilner et al., 1995a; Vilner and Bowen, 2000; Marrazzo et al., 2011b; Zampieri et al., 2016) • Sigma2 (K_i_): 13–58 nM (Hellewell et al., 1994; Vilner and Bowen, 2000; Marrazzo et al., 2011b; Zampieri et al., 2016)	Agonist (Sigma1 and Sigma2/TMEM97)	MTT, LDH release, apoptosis assays	Blocked voltage-activated K+ currents and induced p27^kip1^ levels, inhibition of cell proliferation in some studies by proposed G1 cell cycle arrest. Blocked haloperidol-induced cytotoxicity.	(Brent and Pang, 1995; Moody et al., 2000; Colabufo et al., 2004; Renaudo et al., 2004; Kim and Maher, 2017)
Haloperidol 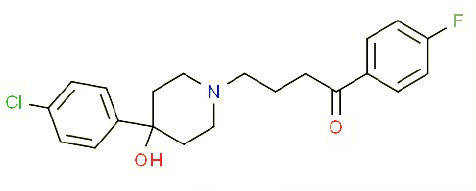	• Sigma1 (K_i_): 1–40 nM (Vilner and Bowen, 1993; Hellewell et al., 1994; Vilner et al., 1995a; Vilner and Bowen, 2000; Choi et al., 2001; Holl et al., 2009a; Holl et al., 2009b; Holl et al., 2009c; Marrazzo et al., 2011a; Marrazzo et al., 2011b; Weber et al., 2014) • Sigma2 (K_i_):12–221 nM Hellewell et al., 1994; Vilner and Bowen, 2000; Choi et al., 2001; Holl et al., 2009a; Holl et al., 2009b; Holl et al., 2009c; Marrazzo et al., 2011a; Marrazzo et al., 2011b; Weber et al., 2014	Antagonist (Sigma1)	MTT, MTS, trypan blue exclusion, apoptosis assays, micrographs of cell morphology changes, colony formation, soft agar assay	Antiproliferative and proapoptotic actions in range of cancer cell lines. Reported to induce unfolded protein response and autophagy. Anticancer actions of haloperidol have been proposed to be both Sigma1- and Sigma2-mediated.	(Brent and Pang, 1995; Vilner et al., 1995a; Moody et al., 2000; Colabufo et al., 2004; Spruce et al., 2004; Wang et al., 2004; Nordenberg et al., 2005; Rybczynska et al., 2008; Megalizzi et al., 2009; Sunnam et al., 2010; Pal et al., 2011; Kim et al., 2012; Schrock et al., 2013; Korpis et al., 2014; Kim and Maher, 2017)
Igmesine 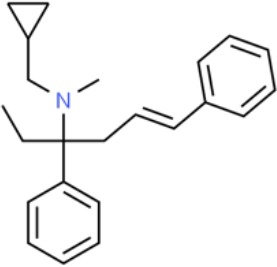	• Sigma1 (IC_50_): 39 nM (Roman et al., 1990)	Agonist (Sigma1)	Trypan blue exclusion, apoptosis assays, cell cycle assays	Inhibited cell proliferation of some cell lines. Blocked voltage-activated K+ currents and induced p27^kip1^ levels, suggesting G1 arrest. Was not cytotoxic and did not induce caspase-mediated apoptosis.	(Renaudo et al., 2004; Renaudo et al., 2007; Gueguinou et al., 2017; Kim and Maher, 2017)
IPAG 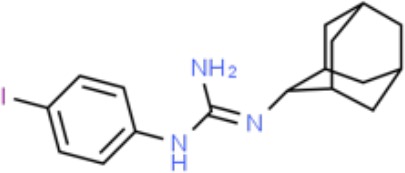	• Sigma1 (K_d_): 3 nM (Wilson et al., 1991; Schrock et al., 2013) • Sigma1 low-affinity site (K_i_): 500–8,000 nM (Brimson et al., 2011)	Antagonist (Sigma1)	Trypan blue exclusion, MTT, MTS, CellTiter-Glo, apoptosis assays, cell cycle, soft agar, colony formation assays, *in vivo* imaging	Selective and potent anticancer activities in range of cancer cell lines, with reported antiproliferative and proapoptotic actions. Induces unfolded protein response and autophagy. Mimics RNAi-mediated knockdown of Sigma1. Triggers lysosomal and proteasomal degradation of cancer promoting signaling proteins including PD-L1, ErbB receptors, and androgen receptor. Multiple high and low-affinity Sigma1-binding sites with distinct activities in intact cancer cells identified. Radiolabeled IPAG tracer used as selective *in vivo* tumor imaging agent.	(Spruce et al., 2004; Megalizzi et al., 2009; Brimson et al., 2011; Kim et al., 2012; Schrock et al., 2013; Kim and Maher, 2017; Thomas et al., 2017; Maher et al., 2018; Gangangari et al., 2019)
PB28 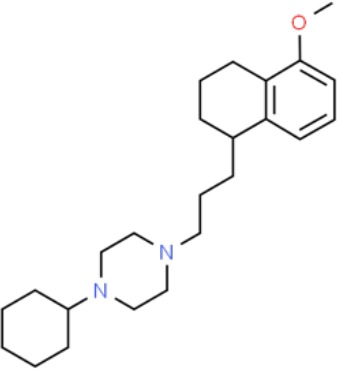	• Sigma1 (K_i_): 10 nM (Azzariti et al., 2006) • Sigma2 (K_i_): 0.28 nM (Azzariti et al., 2006)	Agonist (Sigma2/TMEM97)	MTT, CellTiter-Glo, apoptosis assays, *in vivo* tumor xenografts	Cytotoxic agent that induces ceramide-dependent/caspase-independent apoptosis in part by triggering the production of mitochondrial superoxide radicals. PB28 also reduced P-gp expression on cancer cell lines. Potentiates doxorubicin. Inhibited tumor growth *in vivo.*	(Colabufo et al., 2004; Azzariti et al., 2006; Hornick et al., 2010; Hornick et al., 2012a; Hornick et al., 2012b; Niso et al., 2013a; Korpis et al., 2014; Pati et al., 2017a; Kim and Maher, 2017)
PRE-084 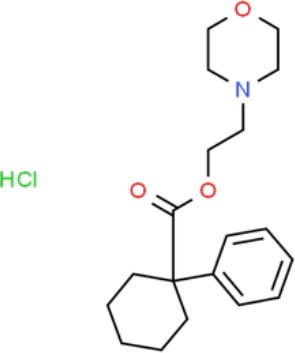	• Sigma1 (K_i_): 53 nM (Garces-Ramirez et al., 2011) • Sigma2 (K_i_): 32,100 nM (Garces-Ramirez et al., 2011)	Agonist (Sigma1)	Trypan blue exclusion, flow cytometry, tumor allografts	Promoted tumor growth in immune competent mouse tumor allograft model by an IL-10-dependent mechanism. No clear evidence of effects on cancer cell proliferation in cell autonomous culture *in vitro* or in xenografts.	(Zhu et al., 2003; Kim et al., 2012; Kim and Maher, 2017)
Rimcazole 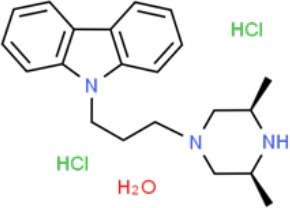	• Sigma1 (K_i_): 406–1,165 nM (Tanaka et al., 1995; Vilner et al., 1995a) • Sigma2 (K_i_): 571–852 nM (Schepmann et al., 2011)	Antagonist (Sigma1)	Trypan blue exclusion, MTT, MTS, CellTiter-Glo, apoptosis assays, cell cycle assays, soft agar colony formation assays, *in vivo* tumor xenografts	Decreased viability, inhibition of cell proliferation, induction of apoptosis. Inhibition of colony formation in 2D colony formation and 3D soft agar assays. HIF1α induction by rimcazole contributes to its anticancer effects. Inhibited tumor growth and cancer cell proliferation in xenograft studies.	(Brent and Pang, 1995; Spruce et al., 2004; Achison et al., 2007; Rybczynska et al., 2008; Rybczynska et al., 2013; Happy et al., 2015; Kim and Maher, 2017)
SA4503 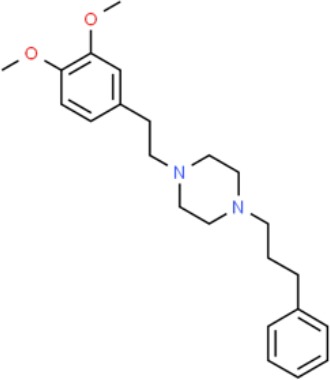	• Sigma1 (K_i_): 4.6 nM (Lever et al., 2006) • Sigma2 (K_i_): 63.1 nM (Lever et al., 2006)	Agonist (Sigma1)	Trypan blue exclusion, confocal microscopy, *in vivo* tumor imaging	Blocks IPAG-induced autophagic degradation of PD-L1 in cancer cells. Promotes PD-L1 cell surface expression on cancer cells. (^11^C)SA4503 development as a tumor imaging agent.	(Ramakrishnan et al., 2013; Kim and Maher, 2017; Maher et al., 2018)
Siramesine 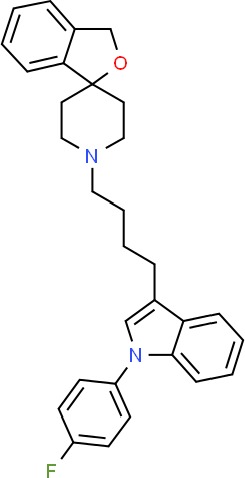	• Sigma1 (K_i_): 10 nM (Niso et al., 2013a) • Sigma2 (K_i_): 13 nM (Niso et al., 2013a)	Agonist (Sigma2/TMEM97)	MTT, MTS, LDH release, apoptosis assays, *in vivo* tumor xenograft studies	Lysosomotropic detergent that triggers lysosomal membrane permeabilization and leakage, increased reactive oxygen species, and apoptotic cell death of cancer cells. MEFs transformed with Src or Ras oncogenes sensitized to siramesine-induced cytotoxicity. Inhibited tumor growth in xenograft studies.	(Ostenfeld et al., 2005; Ostenfeld et al., 2008; Hornick et al., 2010; Zeng et al., 2012; Niso et al., 2013b; Zeng et al., 2014; Kim and Maher, 2017)
SR31747A 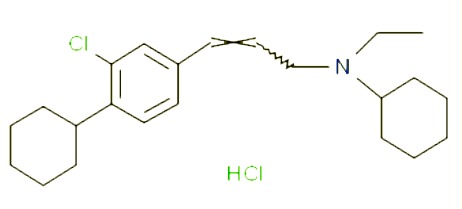	• Sigma1 (K_i_): 3 nM (Laggner et al., 2005)	Antagonist (Sigma1)	MTT, MTS assays, *in vivo* tumor xenografts	Immune modulatory and antiproliferative activities. Inhibited proliferation of range of cancer cell lines. Potentiated tumor growth inhibition of flutamide and tamoxifen in xenograft studies.	(Berthois et al., 2003; Ferrini et al., 2003; Casellas et al., 2004; Kim and Maher, 2017)
SV119 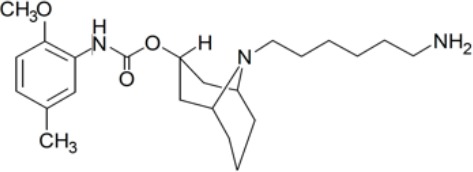	• Sigma1 (K_i_): 1,418 nM (Vangveravong et al., 2006) • Sigma2 (K_i_): 5–8 nM (Vangveravong et al., 2006; Hornick et al., 2010)	Agonist (Sigma2/TMEM97)	MTS, CellTiter-Glo, LDH release, cell cycle assays, apoptosis assays, colony formation, *in vivo* tumor xenografts	Inhibited cancer cell proliferation *in vitro*. Less potent than siramesine. Induced autophagy. SV119 alone induced apoptosis and potentiated cytotoxic and antitumor effects of gemcitabine and paclitaxel *in vitro* and in xenografted tumors *in vivo*.	(Kashiwagi et al., 2007; Kashiwagi et al., 2009; Hornick et al., 2010; Spitzer et al., 2012; Zeng et al., 2012; McDonald et al., 2017)
WC-26 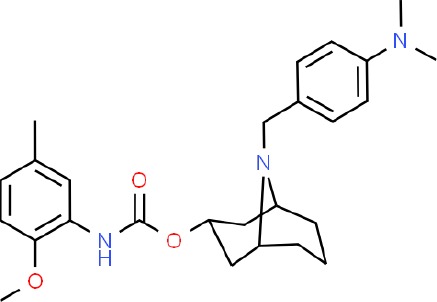	• Sigma1 (K_i_): 1,436 nM 138 • Sigma2 (K_i_): 2.58 nM 138	Agonist (Sigma2/TMEM97)	MTS, MTT, LDH release assay, apoptosis assays, colony formation assay	Inhibited cancer cell proliferation and triggered apoptosis *in vitro*. Induced autophagy. Potentiated doxorubicin-induced cytotoxicity.	(Kashiwagi et al., 2007; Chu et al., 2009; Zeng et al., 2012; McDonald et al., 2017)

As it had remained a pharmacologically defined entity until recently, the elevated expression and levels of Sigma2 have been extrapolated from radioligand binding of cancer cell lines (Bowen, 2000; Wheeler et al., 2000; Zeng et al., 2017). Through pharmacological studies, Sigma2 also has been proposed as a potential drug target in cancer (Wheeler et al., 2000; Crawford and Bowen, 2002; Crawford et al., 2002), and radiotracers with affinity for Sigma2 have been developed as tumor imaging agents (Zeng et al., 2017). The recent identification of Sigma2 as TMEM97 now provides a molecular entity to elucidate the mechanism of action of historical Sigma2 ligands. However, it also raises questions regarding Sigma2 pharmacology in the context of cancer.

There are relatively few publications specifically regarding TMEM97 in cancer. Nevertheless, TMEM97 is reported to be upregulated in cancer cell lines and tumors including esophageal, gastric, colorectal, breast, ovarian surface epithelium (suggesting a role in ovarian cancer), oral squamous, and non-small cell lung cancer (NSCLC) (reviewed in Kayed et al., 2004; Wilcox et al., 2007; Schmit and Michiels, 2018). The pharmacologically defined Sigma2-binding site is reported to be enriched in a broad range of cancer cell lines and solid tumors, including breast and pancreatic (Wheeler et al., 2000; Choi et al., 2001; Hou et al., 2006; Zeng et al., 2007) cancers. However, the reported levels of TMEM97 are not always consistent with those of the pharmacologically defined Sigma2-binding site. For example, Sigma2 binding is elevated in pancreas cancer cell lines, and several published studies demonstrate the potential for Sigma2 ligands as pancreatic cancer therapeutic (Kashiwagi et al., 2007) and imaging agents (Zeng et al., 2017). However, in at least one published study, pancreatic and renal cancers are reported to express low levels of TMEM97 (Kayed et al., 2004). TMEM97 mRNA transcript levels were reported to be highly variable in commonly used pancreatic cancer cell lines with generally low levels of protein (Kayed et al., 2004; Schmit and Michiels, 2018). Elevated TMEM97 expression has been associated with poor clinical outcomes and tumor progression in gastric, colorectal (Moparthi et al., 2007), breast and ovarian (Xiao et al., 2013; Yang et al., 2013), squamous cell lung cancer (SQCLC) (Ding et al., 2016), and non-small cell lung cancer (NSCLC) (Han et al., 2013; Ding et al., 2017). Interestingly, in the latter, high TMEM97 levels correlated with poor patient survival and resistance to platinum-based chemotherapy treatment (Chen et al., 2016; Ding et al., 2017). TMEM97 has been implicated in cancer drug resistance in several reports (Abate et al., 2018).

Furthermore, TMEM97 appears to play the role of tumor suppressor or promotes tumor growth, depending upon the cancer type. TMEM97 has been proposed as a potential tumor suppressor in pancreas (Kayed et al., 2004) and prostate cancer (Ramalho-Carvalho et al., 2018). In contrast, TMEM97 contributes to xenografted tumor growth using glioma and gastric cancer cell line models (Xu et al., 2014; Qiu et al., 2015). In these studies, TMEM97 knockdown in commonly used glioma (U373, U87) and gastric cancer (AGS, BGC-823) cell lines resulted in decreased cell proliferation, migration, and invasion in *in vitro* assays (Xu et al., 2014; Qiu et al., 2015). In contrast to these knockdown studies, Zeng et al. recently published that knockdown and knockout of TMEM97 did not suppress the proliferation or viability of HeLa cells (Zeng et al., 2019). Furthermore, this study proposed that TMEM97 does not mediate Sigma2 ligand–induced cytotoxicity of HeLa cells (Zeng et al., 2019). This study raises important questions regarding our current knowledge of Sigma2 pharmacology in the context of cancer. Considering the apparent context-dependent actions of Sigma2/TMEM97, it will be of interest to further evaluate this approach in a broader range of cancer cell lines.

The recent identification of the Sigma2-binding site as TMEM97 presents an opportunity to merge two fields: for the TMEM97 field to benefit from the decades of medicinal chemistry that has produced a plethora of small-molecule compounds with affinity for Sigma2, and equally, for the Sigma2 field to elucidate the pharmacological mechanism of action of these compounds. It will be interesting to follow the evolution of this subfield over the next several years.

## Putative Agonists and Antagonists of Sigma1 and Sigma2/TMEM97

As it was originally identified as a receptor, small molecules with affinity for Sigma1 and 2, so-called sigma receptor ligands, have been classified as putative agonists and antagonists. These classifications may be inaccurate, as Sigma1 is not a bona fide receptor. Sigma1 has been associated with myriad signaling and transduction systems largely through studies with these ligands (Kruse, 2016; Sabino and Cottone, 2016; Katz et al., 2017; Kim, 2017; Kim and Maher, 2017; Kourrich, 2017; Kruse, 2017; Laurini et al., 2017; Maurice and Goguadze, 2017; Merlos et al., 2017; Pasternak, 2017; Weber and Wunsch, 2017; Zeng et al., 2017). However, Sigma1 has no known intrinsic activity, and a preponderance of evidence suggests that it exerts its actions through allosteric modulation of other proteins and signaling systems (Hayashi and Su, 2007; Maurice and Su, 2009; Kim and Maher, 2017; Pasternak, 2017). Thus, Sigma1 ligands may function as allosteric modulators of protein–protein interactions (PPIs) (Thompson et al., 2012; Cesa et al., 2015; Kim and Maher, 2017; Pricer et al., 2017). In the absence of identified intrinsic activity of the protein itself, the concept of Sigma1 “agonism” and “antagonism” is atypical, such that antagonist actions mimic phenotypes observed in genetic knockdown or knockout animal models (reviewed in Maurice and Su, 2009; Kim and Maher, 2017; Merlos et al., 2017). The term modulator may more accurately define compounds with affinity for Sigma1 (Su et al., 2010; Kim and Maher, 2017). Recently, the oligomeric state of Sigma1 was proposed to be differentially modulated by Sigma1 agonists and antagonists (Gromek et al., 2014; Yano et al., 2018). This was supported by molecular dynamics studies based on the published Sigma1 crystal structure (Schmidt et al., 2018; Yano et al., 2018).

As we have recently published a more comprehensive review of the literature and perspective on Sigma1 biology and Sigma1 pharmacology in the context of cancer elsewhere (Kim and Maher, 2017), in the present review article, we will focus on and expand our discussion of Sigma1 and Sigma2/TMEM97 ligands and their actions in cancer-relevant physiological processes, including cancer cell proliferation, growth, motility, migration, survival, and death (by apoptotic and non-apoptotic mechanisms), as well as protein homeostasis, lipid metabolism, and immune modulation. We also discuss the safety of sigma modulators as well as potential therapeutic benefits in cancer and cancer treatment-associated comorbidities.

Although endogenous ligands for Sigma1 and Sigma2/TMEM97 have not been clearly established, sigma receptor ligands were initially defined as agonists and antagonists based on rodent behavior assays (discussed in Cobos EJ et al., 2008; Maurice and Su, 2009; Katz et al., 2017; Kim, 2017; Kim and Maher, 2017; Kruse, 2017; Maurice and Goguadze, 2017; Merlos et al., 2017; Pasternak, 2017; Weber and Wunsch, 2017), wherein synthetic Sigma1 agonists generally promoted the actions of other drugs, such as neurosteroids, cocaine, and amphetamines. Conversely, Sigma1 antagonists either produced no behavioral changes (Matsumoto et al., 2001; Maurice and Goguadze, 2017) or attenuated stimulant triggered behaviors (Maurice et al., 1998; McCracken et al., 1999). For example, the neurosteroid pregnenolone and dehydroepiandrosterone, both of which have affinity for Sigma1, were neuroprotective and thus classified as Sigma1 agonists, whereas progesterone blocked their neuroprotective effects and thus was classified as a Sigma1 antagonist (Maurice et al., 1998). These studies are reviewed and discussed in detail elsewhere (Cobos EJ et al., 2008; Maurice and Su, 2009; Katz et al., 2017; Kim, 2017; Kim and Maher, 2017; Kruse, 2017; Maurice and Goguadze, 2017; Merlos et al., 2017; Pasternak, 2017; Weber and Wunsch, 2017).

Inhibitions of cancer cell proliferation and cell viability have been considered measures of Sigma1 inhibition (putative antagonism), and this is largely consistent with the effects of Sigma1 ablation/knockdown on cancer cells (reviewed in 15). However, as we discuss below, the distinction between putative agonists and antagonists does not strictly apply.

There remains no established biochemical or molecular mechanism of action to clearly define Sigma1 agonist/activator and antagonist/inhibitor activity. However, recently, oligomerization has been proposed as a readout of differential Sigma1 agonist/activator *versus* antagonist/inhibitor activity (Gromek et al., 2014; Yano et al., 2018). This is consistent with a role for Sigma1 as an allosteric modulator of protein–protein interactions and associated protein signaling.

The definition of Sigma2/TMEM97 agonist and antagonist remains unclear. Zeng et al. have proposed that Sigma2 selective compounds with cancer cell cytotoxic effects on cancer cells should be classified as agonists. This is based on the cytotoxicity of siramesine, which the authors cite as a commonly accepted Sigma2 agonist (Zeng et al., 2014). Using this approach, the authors have categorized Sigma2 ligands as agonists, partial agonists, and antagonists (Zeng et al., 2014). However, these do not provide molecular basis for pharmacological mechanism of action of Sigma2 ligands.

## Actions of Putative Activators/Agonists and Inhibitors/Antagonists of Sigma1 and Sigma2/TMEM97 in Standard Preclinical Models of Cancer

Much of our knowledge regarding Sigma1 and Sigma2/TMEM97 in tumor biology is derived from studies with synthetic compounds. Several prototypic Sigma1 and Sigma2/TMEM97 compounds are reported to influence cancer cell survival, proliferation, growth, adhesion, motility, and protein homeostasis pathways, thereby suggesting a potentially broad range of therapeutic opportunities for targeting these proteins (reviewed in Kim and Maher, 2017). Several key prototypic compounds are highlighted in **Table 1**.

### Antiproliferative and Proapoptotic Actions of Sigma1 Inhibitors/Antagonists

In preclinical laboratory models of cancer, Sigma1 inhibition or putative antagonism is generally associated with inhibition of cancer cell proliferation and viability. Interestingly, Sigma1 putative antagonists/inhibitors as originally defined by behavioral endpoints have generally correlated with inhibition of cancer cell proliferation and in some cases induction of apoptosis (Colabufo et al., 2004; Spruce et al., 2004). Importantly, this is consistent with the general proliferation, growth, and survival inhibiting effects of Sigma1 RNAi knockdown (reviewed in Kim and Maher, 2017). A detailed and extensive review and discussion of the antiproliferative and proapoptotic actions of sigma modulators is provided elsewhere (Kim and Maher, 2017). Importantly, the *in vivo* anti-tumor efficacy of several prototypic Sigma1 antagonists/inhibitors has been reported, highlighting their drug-like properties and potential for drug development. Furthermore, most of these studies report efficacious tumor growth inhibition with minimal toxicity in mouse models (reviewed in Kim and Maher, 2017).

### Proliferative and Prosurvival Actions of Sigma1 Activators/Agonists

In most *in vitro* cancer biology studies, Sigma1 agonists/activators have been used to observe pharmacological competition to confirm Sigma1 selective actions. Typically, Sigma1 agonists/activators appear to have no effect on cell proliferation and tumor growth (Kim and Maher, 2017). The common prototypic agonists used to this end include (+)-pentazocine, (+)-SKF10047, PRE-084, 4-(N-benzylpiperidin-4-yl)-4-iodobenzamide (4-IBP), and SA4503. In some cases, these putative agonists/activators are reported to promote cancer cell proliferation and tumor growth (reviewed in 15). However, some publications report the contrary that some of these same compounds inhibit cell proliferation and trigger cell cycle arrest (Megalizzi et al., 2007; Megalizzi et al., 2009). It is difficult to reconcile these discrepancies. However, the notion of agonist/activator and antagonist/inhibitor classifications may be inaccurate, and what these classifications mean in the context of cancer cell biology remains unclear.

### Cytotoxic actions of Sigma2/TMEM97 Agonists/Activators

Interestingly, Zeng et al. reported that neither Sigma2/TMEM97 nor PGRMC1 (which was originally identified as the Sigma2-binding site Xu et al., 2011; Abate et al., 2015; Zeng et al., 2016; Pati et al., 2017b)-mediated Sigma2 ligand–induced cytotoxicity (Zeng et al., 2019). Based on this surprising discovery, the authors propose a closer evaluation of the mechanisms underlying Sigma2 ligand–induced cytotoxicity (Zeng et al., 2019). Thus, the anti-cancer mechanism of action of putative Sigma2 selective compounds remains unclear.

### Combined Sigma1 Inhibitors/Antagonists and Sigma2/TMEM97 Agonist/Activators

Most putative sigma receptor ligands have affinity for both Sigma1 and Sigma2/TMEM97, albeit with differences in subtype-binding affinity (reviewed in 15 and **Table 1**). It has been proposed that the antiproliferative and proapoptotic activities of these compounds may involve a combination of Sigma1 antagonism/inhibition and Sigma2 agonism (Zeng et al., 2014). However, when this concept was proposed, Sigma2 was still a pharmacologically defined entity as the identity of Sigma2 has been controversial (Abate et al., 2015; Pati et al., 2017b). Furthermore, the definition of Sigma2 agonism is unclear. The recent identification of TMEM97 as Sigma2 (Alon et al., 2017) should accelerate the elucidation of the pharmacological mechanism of action of putative Sigma2 ligands. Furthermore, more data are needed to clarify the roles of TMEM97 alone and in relation to Sigma1 in cancer pharmacology (Schmit and Michiels, 2018).

## Cellular Pathways, Processes, and Signaling Systems Engaged by Modulation of Sigma1 and Sigma2/TMEM97

Much of the sigma ligand–related cancer literature includes endpoint readouts of cell proliferation and cell death. A growing body of literature reports the cellular pathways and processes engaged by modulation of Sigma1 and what we now know to be Sigma2/TMEM97. The cellular and molecular mechanisms underlying these effects remain poorly understood. However, several themes are emerging, implicating Sigma1 in the modulation of protein and lipid homeostasis, autophagy, and ion channel regulation (Kim and Maher, 2017).

### Regulators of Protein and Lipid Homeostasis

Cancer cells are associated with aberrant growth and metabolism, resulting in increased demand for protein production, corresponding membrane biogenesis, and *de novo* synthesized fatty acids as an energy source. This renders tumors particularly dependent on factors that maintain homeostasis of protein and lipid metabolism (Ma and Hendershot, 2004a; Ma and Hendershot, 2004b; Denoyelle et al., 2006; Jones and Thompson, 2009; Luo et al., 2009; Sonenberg and Hinnebusch, 2009). Emerging data suggest that Sigma1 is a multifunctional chaperone or scaffolding protein involved in maintaining ER protein homeostasis and supporting the increased demand for secretory pathway protein synthesis associated with tumor growth (Kim and Maher, 2017). Pharmacological modulation of Sigma1 in cancer cells has been shown to alter the protein synthesis, post-translational modification, trafficking, and degradation of cancer promoting proteins (Hayashi and Su, 2007; Crottes et al., 2011; Kim et al., 2012; Schrock et al., 2013; Crottes et al., 2016; Thomas et al., 2017). In this respect, Sigma1 ligands are reminiscent of proteostasis regulators (Powers et al., 2009).

Proliferation is associated with regulation of growth, which involves the increase in biomass essential for successful cell doubling (Luo et al., 2009). Sigma1 modulators can be used to control biomass of cancer cells *via* regulation of protein translation (Kim et al., 2012) and protein degradation *via* ubiquitin proteasome system (UPS)–mediated and autophagosomal degradation mechanisms (Schrock et al., 2013; Thomas et al., 2017; Maher et al., 2018). Sigma1 modulators have also been shown to impact cellular pathways driving cell growth, such as PI3K/Akt/mTOR (Spruce et al., 2004; Kim et al., 2012; Zeng et al., 2012). Sigma1 inhibition did this in a PTEN-independent manner (Kim et al., 2012; Schrock et al., 2013; Kim and Maher, 2017; Thomas et al., 2017).

### Unfolded Protein Response (UPR) and Autophagy

Sigma1 antagonists/inhibitors have been shown to trigger the unfolded protein response (UPR) in cancer cells. Schrock et al. evaluated a panel of structurally diverse compounds with affinity for Sigma1 and found that a subset of prototypic Sigma1 antagonists/inhibitors–induced UPR and autophagy in a range of cancer cell lines in a dose- and time-responsive manner (Schrock et al., 2013). Of note, these effects were reversible upon washout of the compound, as demonstrated with IPAG, a selective high-affinity Sigma1 antagonist/inhibitor (Kim et al., 2012; Schrock et al., 2013; Thomas et al., 2017). If the basis of Sigma1 ligand action is modulation of PPI, then the reversal of these actions following compound removal suggests that these Sigma1 antagonist/inhibitor-mediated effects require high occupancy of Sigma1 and that disruption of Sigma1 PPIs requires continuous target engagement. Consistent with this notion, Schrock et al. demonstrated that IPAG induced apoptosis, but only after extended treatment, suggesting that an apoptosis trigger occurs when a certain threshold is surpassed (Schrock et al., 2013). These studies suggest that Sigma1 modulators may be useful as pharmacological regulators of cancer cell protein homeostasis (Kim et al., 2012; Schrock et al., 2013; Thomas et al., 2017).

### Cholesterol/Lipid Binding

There are preliminary but intriguing data regarding a potential role for sigma proteins in lipid metabolism. As discussed above, cancer cells are particularly dependent on factors that maintain lipid homeostasis (Ma and Hendershot, 2004a; Ma and Hendershot, 2004b; Denoyelle et al., 2006; Jones and Thompson, 2009; Luo et al., 2009; Sonenberg and Hinnebusch, 2009) due to rapid growth and corresponding abnormal metabolism. Although a role for Sigma1 in lipid metabolism has not been established, a few studies have implicated a physiological role for Sigma1 and Sigma2/TMEM97 in cholesterol dynamics.

Sigma1 has been hypothesized to contain two cholesterol-binding domains (CBD) adjacent to the Sigma1 ligand–binding site (Palmer et al., 2007; Schmidt et al., 2016). Sigma1 has been proposed to contribute to remodeling of cholesterol rich lipid rafts, and in one report, Sigma1 binding to cholesterols was inhibited by (+)-SKF10047 (Palmer et al., 2007). Thus, disruption of Sigma1 may alter the cholesterol content of the surrounding lipid bilayer, and the subsequent remodeling of lipid rafts would disrupt the signaling complexes dependent on these stabilizing and organizing platforms (Simons and Toomre, 2000; Aydar et al., 2002; Aydar et al., 2004; Jacobson et al., 2007; Palmer et al., 2007; Balasuriya et al., 2014).

Choline was recently proposed as an endogenous Sigma1 agonist/activator (Brailoiu et al., 2019). Interestingly, choline is a lipid precursor associated with aggressive prostate cancer (Richman et al., 2012; Zadra et al., 2013; Pavlova and Thompson, 2016). Clinically, choline intake has been associated with an increased risk of lethal prostate cancer (Richman et al., 2012). Altogether, these data provide evidence of a role for Sigma1 in cancer cell lipid metabolism. This is an interesting and emerging area of research that remains poorly understood.

Expression of Sigma2/TMEM97, along with several cholesterol biosynthesis genes, was reported to be induced by progesterone in ovarian surface epithelial (OSE) cells, the cell type from which ovarian cancer often derives. In this context, upregulation of TMEM97 in OSE cells by progesterone was proposed to protect against the development of ovarian cancer (Wilcox et al., 2007).

Recently, Sigma2/TMEM97 was shown to interact with low-density lipoprotein (LDL) receptor and to be involved in LDL uptake (Riad et al., 2018). This is consistent with the published role of TMEM97 in cholesterol homeostasis (see above).

### Cell Motility, Migration, and Adhesion

Sigma1 RNAi knockdown and treatment with (+)-SKF10047, a putative agonist/activator, have been shown to disrupt cancer cell motility, migration, and adhesion *in vitro* by regulating cell surface expression of β-integrin (Aydar et al., 2006; Palmer et al., 2007). The correlation between Sigma1 knockdown and (+)-SKF10047 treatment is surprising and is inconsistent with the definition of (+)-SKF10047 as an agonist/activator. Treatment of cancer cells *in vitro* with (+)-SKF10047 decreased Sigma1-β-integrin association in lipid raft fractions and resulted in Sigma1 dissociation from lipid rafts (Palmer et al., 2007). Others have reported that 4-IBP and haloperidol inhibited cell migration and motility of multiple cancer cell lines including human glioblastoma (U373-MG), melanoma (C32), NSCLC (A549), and prostate cancer (PC3) (Megalizzi et al., 2007; Rybczynska et al., 2008; Megalizzi et al., 2009). These *in vitro* data have been used as evidence to suggest that Sigma1 plays a role in metastasis (Aydar et al., 2006; Palmer et al., 2007; Aydar et al., 2016). However, whether Sigma1 and its pharmacological modulators impact metastasis in *in vivo* models remains unclear. No studies to establish the role of Sigma1 in metastasis *in vivo* have been reported.

### Allosteric Regulation of Oncogenic Driver Proteins and Signaling Axes

The protein homeostasis regulating properties of Sigma1 ligands may be exploited to modulate oncogenic protein signaling. The actions of Sigma1 modulators are largely defined by their associated signaling systems. Emerging data support the notion that Sigma1 is an allosteric modulator/regulator of signaling proteins and signaling axes. Sigma1 ligands can selectively regulate the stability, trafficking, and signaling of oncogenic driver proteins (Kim and Maher, 2017; Thomas et al., 2017; Maher et al., 2018).

Recently, Sigma1 was found to regulate aberrant androgen receptor (AR) activity and stability in prostate cancer cells (Thomas et al., 2017). The objectives of this study were to better understand the interaction of Sigma1 with an oncogenic protein, in this case AR, and to determine the potential therapeutic value of targeting Sigma1 in this context (Thomas et al., 2017). Sigma1 physically associated with AR, and pharmacological inhibition of Sigma1 blocked AR nuclear translocation and suppressed its transcriptional activity in response to androgen (5α-dihydrotestosterone [5α-DHT]). It also triggered the proteasomal degradation of AR and constitutively active AR splice variants (ARVs). Sigma1 also interacts with ErbB receptors, and the prototypic Sigma1 antagonist/inhibitor dose-responsively suppressed ErbB-2 and -3 receptor protein levels (Thomas et al., 2017).

### Ion channels in cancer

Several studies have shown Sigma1 ligand modulation of ion channel activity in cancer cell lines (Renaudo et al., 2004; Renaudo et al., 2007; Wu and Bowen, 2008; Crottes et al., 2011; Balasuriya et al., 2014; Crottes et al., 2016; Gueguinou et al., 2017). This has been reviewed extensively elsewhere (Crottes et al., 2013; Kim and Maher, 2017). Interestingly, Sigma1 putative agonist/activators were used in many of these studies to support the notion that Sigma1 modulation of ion channel activities can suppress cancer cell proliferation, adhesion, motility, and migration (Renaudo et al., 2004; Renaudo et al., 2007; Wu and Bowen, 2008; Crottes et al., 2011; Balasuriya et al., 2014; Crottes et al., 2016; Gueguinou et al., 2017). Very recently, choline was proposed as an endogenous Sigma1 agonist/activator based on its ability to bind Sigma1 and mimic other putative Sigma1 agonists by potentiating Ca^2+^ signals evoked by inositol triphosphate receptors (IP_3_Rs) (Brailoiu et al., 2019).

## Immune Modulation

A growing body of evidence demonstrates that inhibition of Sigma1 can suppress growth, decrease proliferation, and induce apoptosis in multiple cancer cell lines through regulation of cell-intrinsic signaling in cancer cells (Kim and Maher, 2017). However, the impact of targeting Sigma1 may extend beyond regulation of cell-intrinsic signaling proteins and pathways. Several publications describe the immunomodulatory properties of Sigma1 ligands (Bourrie et al., 1995; Carayon et al., 1995; Derocq et al., 1995; Bourrie et al., 1996; Carayon et al., 1996; Bourrie et al., 2002; Zhu et al., 2003; Bourrie et al., 2004; Gardner et al., 2004). Sigma1 agonists/activators PRE-084 and (+)-SKF10047 stimulate production of immunosuppressive cytokines that block the host antitumor immune response in the tumor microenvironment (reviewed in Kim and Maher, 2017). In at least one reported study, PRE-084 and (+)-SKF10047 induced the extracellular secretion of IL-10, TGF-β, and PGE2, while decreasing IFN-γ at the tumor site (Zhu et al., 2003). The increase in TGF-β production or secretion was observed only in tumor-bearing mice and was absent in normal, non-tumor bearing mice (Zhu et al., 2003). PRE-084 has been shown to promote tumor growth in a syngeneic lung cancer (L1C2 murine alveolar cell carcinoma) model in part by inducing IL-10 at the tumor site (Zhu et al., 2003). Co-treatment with PRE-084 and BD1047 (putative Sigma1 antagonist or inhibitor) blocked the tumor growth promoting effects of PRE-084, showing that this effect is Sigma1-mediated. An anti-IL-10 antibody (JES-2A5) blocked the tumor growth promoting effect of PRE-084, showing that tumor growth is at least partially dependent on IL-10. The immunomodulatory or tumor growth effects of BD1047 alone were not evaluated in this study (Zhu et al., 2003). Thus, these studies did not determine whether putative Sigma1 antagonists/inhibitors can mediate antitumor immune responses.

Recently, it was discovered that the stability, trafficking, and activity of programmed death-ligand 1 (PD-L1, alternately named B7-H1, CD274) could be differentially modulated by SA4503 (Sigma1 agonist/activator) and IPAG (Sigma1 antagonist/inhibitor) (Maher et al., 2018). Sigma1 inhibition by IPAG caused the autolysosomal degradation of PD-L1 in PC3 (hormone-insensitive prostate cancer) and MDA-MB-231 (triple-negative breast cancer) cell lines and reduced the levels of functional PD-L1 on the surface of the cells (Maher et al., 2018). Knockdown of Sigma1 by shRNA also reduced PD-L1 levels, showing consistency with the effects of the Sigma1 antagonist/inhibitor IPAG. When the Sigma1 agonist/activator SA4503 was applied alone, the surface levels of PD-L1 increased. When SA4503 was applied with IPAG, the IPAG-mediated decrease of PD-L1 levels was blocked, displaying Sigma1 selective activity (Maher et al., 2018). Induction of PD-L1 by interferon gamma was also blocked by IPAG (Maher et al., 2018). This report demonstrates that PD-L1 production and activity can be regulated by Sigma1 modulation either directly through cell-intrinsic mechanisms or indirectly by immune response–induced cytokine-mediated feedback loops. Thus, Sigma1 ligands may regulate the tumor immune microenvironment. These lines of evidence warrant studies to determine antitumor immunity activity induced by Sigma1 modulation.

## Safety of Sigma Modulation

We previously reviewed clinical and preclinical evidences demonstrating that the on-target actions of Sigma1 modulators do not induce adverse effects (Kim and Maher, 2017). Since then, clinical evidence in support of the safety of Sigma2 modulation/inhibition has been reported (Grundman et al., 2019). The safety and efficacy of Sigma1 modulation are also being evaluated in human clinical trials of S1RA (Sigma1 selective antagonist/inhibitor, also known as E-52862). Proof of concept for the safety and efficacy of Sigma1 modulation has been reviewed extensively elsewhere (Abadias et al., 2013; Kim and Maher, 2017; Merlos et al., 2017; Bruna et al., 2018; Grundman et al., 2019).

## Cancer-Associated Pain

The role of Sigma1 in pain has been studied for decades (reviewed in Pasternak, 2017). However, there are relatively few published studies focused on the utility of sigma receptor ligands in cancer-associated pain. Although a role for sigma receptors in cancer pain remains poorly understood, emerging evidence suggests that Sigma1 selective drugs such as S1RA/E-52862, which is in clinical trials to assess its ability to produce non-opioid analgesia (see above), may be effective agents in this space. Recently, an exploratory randomized, double-blind, placebo-controlled phase II clinical trial generated preliminary proof of concept that treatment with S1RA could mitigate oxaliplatin-induced peripheral neuropathy in patients with colorectal cancer receiving FOLFOX treatment (Bruna et al., 2018). In this hypothesis generating study, intermittent treatment with the Sigma1 antagonist/inhibitor was associated with reduced acute oxaliplatin–induced peripheral neuropathy and allowed patients to be exposed to higher doses of oxaliplatin. Furthermore, the Sigma1 antagonist/inhibitor showed an acceptable safety profile (Bruna et al., 2018). The authors explain that this study, although exciting, must be confirmed broadly to be certain of the protective effects against acute and severe cumulative neuropathy. These studies raise an important question regarding the multiple properties of Sigma1 antagonists/inhibitors. Can a small-molecule Sigma1 antagonist/inhibitor that shows antineoplastic capabilities also be used to manage cancer-associated pain? To date, no clinically used compounds exhibiting these Sigma1 pharmacology properties have been reported.

## Conclusions and Perspectives

Over the past several decades, the story of sigma receptors has undergone many twists and turns, and this is reflected in the broad and complex literature. The field is rapidly evolving, and some of the anchor pieces of the sigma receptor puzzle (Kim, 2017; Kim and Maher, 2017) are starting to emerge. It is now evident that Sigma1 is not a traditional receptor or signaling protein. In the context of cancer, Sigma1 appears to function as a scaffold or chaperone, a component of the cancer cell support machinery. Sigma2 has long remained a pharmacologically defined entity and was recently identified as Sigma2/TMEM97 (Alon et al., 2017); however, important questions remain regarding inconsistencies between the traditional Sigma2 radioligand–binding site and TMEM97 (addressed above).

From a pharmacological perspective, Sigma1 appears to be an allosteric modulator of multiple signaling systems. The increasing number of Sigma1 interacting proteins implicates this protein in a variety of pathophysiological roles. Yet, published *SIGMAR1* KO mice are viable without overt phenotype, at least under routine animal husbandry conditions. Several tumor xenograft studies report absence of measurable adverse effects at efficacious doses (Kim and Maher, 2017). Clinically, a recent phase I trial with a putative Sigma1 inhibitor/antagonist demonstrated proof of concept that such drugs can be safe (see above). A recent phase I trial of a putative Sigma2/TMEM97 targeting compound too was reported to be well tolerated (Grundman et al., 2019). Altogether, sigma drugs appear to elicit distinct actions at Sigma1 and Sigma2/TMEM97 in physiological compared to pathophysiological contexts. These distinct responses may reflect the tissue and disease context-dependent composition of Sigma1-associated multiprotein complexes. An important question is whether Sigma1 and Sigma2/TMEM97 protein complexes change composition, localization, protein–protein interaction dynamics, and dependencies with disease.

An essential missing piece of the sigma puzzle is a clear definition of drug molecular mechanisms of action that translate into downstream physiological response and tumor promoting as well as inhibiting actions of Sigma1 modulation. Recent reports demonstrate Sigma1 compounds can trigger differential changes in Sigma1 oligomerization status corresponding to putative inhibition/antagonism and activation/agonism (Gromek et al., 2014; Yano et al., 2018), and these changes may be responses to corresponding differential conformational shifts based on putative antagonist and agonist status (Kim and Pasternak, 2018; Schmidt et al., 2018). These molecular mechanism studies also show that ligand binding of Sigma1 is a complex, multistep process (Kim and Pasternak, 2018; Schmidt et al., 2018). These initial studies will require further validation and expansion. Breakthroughs in understanding the role of sigma proteins in cancer and the value of sigma targeting agents in cancer and establishing meaningful structure-activity relationships for drug discovery and development will require more systematic and in-depth analyses of intracellular signaling cascades and pathways that connect compound molecular mechanism of action to physiological response. In this respect, the field of sigma proteins in the context of cancer is still relatively under explored and in its early stages. There is a significant need to evaluate different, more sophisticated *in vitro* and *in vivo* experimental cancer models to accurately measure physiological impact and correlation to anti-tumor response.

Targeting Sigma1 and Sigma2/TMEM97 to treat cancer would be highly novel approaches. The multifunctionality and apparent disease-dependent actions of these drug targets offer new therapeutic opportunities. The challenge will be to understand how to modulate them in a physiological and pathophysiological context-dependent manner.

## Author Contributions

HO, CS, and FK contributed to the writing and editing of the manuscript.

## Conflict of Interest

The authors declare that the research was conducted in the absence of any commercial or financial relationships that could be construed as a potential conflict of interest.
